# Thermohydraulic analysis of covalent and noncovalent functionalized graphene nanoplatelets in circular tube fitted with turbulators

**DOI:** 10.1038/s41598-022-22315-9

**Published:** 2022-10-21

**Authors:** Hai Tao, Omer A. Alawi, Omar A. Hussein, Waqar Ahmed, Ali H. Abdelrazek, Raad Z. Homod, Mahmoud Eltaweel, Mayadah W. Falah, Nadhir Al-Ansari, Zaher Mundher Yaseen

**Affiliations:** 1 College of Artificial Intelligence, Nanchang Institute of Science and Technology, Nanchang, China; 2 School of Computer and Information, Qiannan Normal University for Nationalities, Duyun, Guizhou 558000 China; 3 Institute for Big Data Analytics and Artificial Intelligence (IBDAAI), Universiti Teknologi MARA, 40450 Shah Alam, Selangor Malaysia; 4 Department of Thermofluids, School of Mechanical Engineering, Universiti Teknologi Malaysia (UTM), Skudai, 81310 Johor Bahru, Malaysia; 5 Petroleum system control engineering department, College of Petroleum Processes Engineering, Tikrit University, Tikrit, Iraq; 6 Takasago i-Kohza, Malaysia-Japan International Institute of Technology, Universiti Teknologi Malaysia, Kuala Lumpur, Malaysia; 7 Department of Oil and Gas Engineering, Basrah University for Oil and Gas, Basrah, Iraq; 8 School of Physics, Engineering and Computer Science, University of Hertfordshire, Hatfield, AL10 9AB UK; 9 Building and construction techniques engineering department, AL-Mustaqbal University College, Hillah, 51001 Iraq; 10 Civil, Environmental and Natural Resources Engineering, Lulea University of Technology, 97187 Lulea, Sweden; 11 Civil and Environmental Engineering Department, King Fahd University of Petroleum and Minerals, Dhahran, 31261 Saudi Arabia

**Keywords:** Fluid dynamics, Graphene, Nanoscale materials, Theory and computation

## Abstract

Covalent and non-covalent nanofluids were tested inside a circular tube fitted with twisted tape inserts with 45° and 90° helix angles. Reynolds number was 7000 ≤ Re ≤ 17,000, and thermophysical properties were assessed at 308 K. The physical model was solved numerically via a two-equation eddy-viscosity model (SST k-omega turbulence). GNPs-SDBS@DW and GNPs-COOH@DW nanofluids with concentrations (0.025 wt.%, 0.05 wt.% and 0.1 wt.%) were considered in this study. The twisted pipes' walls were heated under a constant temperature of 330 K. The current study considered six parameters: outlet temperature, heat transfer coefficient, average Nusselt number, friction factor, pressure loss, and performance evaluation criterion. In both cases (45° and 90° helix angles), GNPs-SDBS@DW nanofluids presented higher thermohydraulic performance than GNPs-COOH@DW and increased by increasing the mass fractions such as 1.17 for 0.025 wt.%, 1.19 for 0.05 wt.% and 1.26 for 0.1 wt.%. Meanwhile, in both cases (45° and 90° helix angles), the value of thermohydraulic performance using GNPs-COOH@DW was 1.02 for 0.025 wt.%, 1.05 for 0.05 wt.% and 1.02 for 0.1 wt.%.

## Introduction

### Study background knowledge and motivation

Heat exchangers are thermal devices used to transport heat during cooling and heating operations^1^. Heat exchanger thermohydraulic performance increases heat transfer coefficients and lowers working fluid resistance. Some heat transfer enhancement techniques have been developed, including turbulence promoters^2–11^ and nanofluids^12–15^. Due to its simplicity of maintenance and low cost, twisted tape insertion is one of the most successful ways of enhancing heat transfer in a heat exchanger^7,16^.

### Implemented literature review on nanofluids-based twisted pipes

In a series of experimental and computational research, the hydrothermal characteristics of a mixture of nanofluids and a heat exchanger with twisted tape inserts were investigated. Experimental work explored the hydrothermal properties of three different metallic nanofluids (Ag@DW, Fe@DW, and Cu@DW) within a heat exchanger with spiky twisted tapes (STT)^17^. The heat transfer coefficient of STT has gone up 11 and 67% compared to the basic pipe. The SST arrangement was the best cost-efficient based on the performance factor, with the parameters of α = β = 0.33. Furthermore, n increase of 18.2% was observed using Ag@DW, even though the largest increase in pressure loss was just 8.5%. The heat transfer and pressure loss physical characteristics in a concentric tube with and without wire coil (WC) turbulators were explored using turbulent forced convection Al_2_O_3_@DW nanofluid flow^18^. Maximum average Nusselt number (Nu_avg_) and pressure loss were seen under the Re = 20,000 when the pitch wire coil = 25 mm and 1.6 volume%-Al_2_O_3_@DW nanofluids. Laboratory studies were also carried out to investigate the heat transfer and pressure loss features of graphene oxide (GO@DW) nanofluids flowing through a basic circular tube with WC inserts^19^. According to the results, 0.12 volume%-GO@DW raised the convective heat transfer coefficient by about 77%. An additional experimental study developed (TiO_2_@DW) nanofluid, examining thermo-hydraulic performances of dimpled tubes fitted having twisted tape inserts^20^. The greatest thermo-hydraulic efficiency of 1.258 was achieved using 0.15 volume%-TiO_2_@DW in a tilted 45°-dimple and embedded with a twisted tape ratio of 3.0. Single-phase and two-phase (mixed) simulation models solved the CuO@DW nanofluid flow and heat transfer in the various solid concentrations (1–4% volume%)^21^. The maximum thermal efficiency in a tube with one twisted tape insertion was 2.18, but it was 2.04 in a tube with two twisted tape insertions on the same terms (two-phase model, Re = 36,000 and 4 volume%). The non-Newtonian turbulent nanofluid flow of Carboxymethyl cellulose (CMC) and Copper oxide (CuO) was examined in a basic pipe and a pipe having twisted insertions^22^. Nu_avg_ demonstrated improvements like 16.1% (for basic pipe) and 60% [for a twisted pipe with a ratio of (H/D = 5)]. Frequently, the smaller twisted tape ratio has established a higher friction factor. An experimental study examined the influences of pipe having twisted tape (TT) and wire coil (WC) on the heat transfer and friction factor properties using CuO@DW nanofluid^23^. Using 0.3volume%-CuO@DW at Re = 20,000 enhanced the heat transfer up to its maximum value of 44.45% in a WC-2 tube. Furthermore, by applying twisted tape and wire coil insertions under the same boundary conditions, the friction factors increased by 1.17-times and 1.19-times compared to DW. In general, the thermal performance factor of nanofluids with wire coil insertions was better than for twisted tape insertions. The overall performance of turbulent (MWCNTs@DW) nanofluid flow was examined inside a horizontal pipe with coiled wire inserted^24^. All cases had a thermal performance parameter > 1, indicating that combining nanofluids with wire coil insertions improved heat transfer without consuming pumping power. Experiments under turbulent Al_2_O_3_ + TiO_2_@DW nanofluid flow conditions were carried out on hydrothermal properties in a double-tube heat exchanger having various modified V-cuts twisted tape (VcTT) insertions^25^. Nu_avg_ was enhanced significantly by the percentage of 132%, and the friction factor was up to 55% when compared to DW in a basic pipe. Also, the exergetic effectiveness of nanocomposite Al_2_O_3_ + TiO_2_@DW was discussed within a double pipe heat exchanger^26^. They found in their research that employing Al_2_O_3_ + TiO_2_@DW and TT increased the exergy efficiency relative to DW. In a concentric tube heat exchanger having a VcTT turbulator, Singh and Sarkar^27^ used phase change material (PCM) dispersed mono/nanocomposite nanofluids (Al_2_O_3_@DW with PCM and Al_2_O_3_ + PCM). They reported that heat transfer and pressure loss increased when the twisting ratio decreased and nanoparticle concentration increased. More heat transfer and pressure loss were achieved with a larger V-cut depth ratio or a lower width ratio. Furthermore, graphene–platinum (Gr-Pt) was applied to examine the thermal, frictional, and total entropy production rates in tubes having 2-TT insertions^28^. Their study noted that less percentage of (Gr-Pt) significantly decreased the thermal entropy formation than relatively increased frictional entropy development. Al_2_O_3_@MgO hybrid nanofluid and tapered WC can be regarded as a good mix because of the enhanced (h/Δp) ratio to improve the hydrothermal properties of a double pipe heat exchanger^29^. A numerical model was used to solve the exergo-economic environmental effectiveness of heat exchanger having various tripartite hybrid nanofluids (THNF) (Al_2_O_3_ + Graphene + MWCNTs) suspended in DW^30^. The combination of dimpled twisted turbulator insert (DTTI) and (Al_2_O_3_ + Graphene + MWCNTs) was desired because its performance assessment criteria (PEC) was in the range of 1.42–2.35.

### Research objectives

So far, very little attention has been paid to the role of covalent and non-covalent functionalization on hydraulic flow in thermal fluids. The specific objective of this study was to compare the thermal–hydraulic performance of (GNPs-SDBS@DW) and (GNPs-COOH@DW) nanofluids within twisted tape inserts with 45-degree and 90-degree helix angles. The thermophysical properties were measured at T_in_ = 308 K. Meanwhile, three mass fractions were taken into consideration during the comparison such as (0.025 wt.%, 0.05 wt.% and 0.1 wt.%). The shear stress transport (SST k-ω) model in three-dimensional turbulence was used to solve the thermohydraulic performance. As a result, by proving Thermal–Hydraulic Performance and Optimization the practical working fluids in such engineering systems, this study offers a significant contribution to research on the positive properties (heat transfer) and negative properties (frictional pressure drop).

## Numerical methodologies

### Physical model and numerical method

The base configuration is a smooth pipe (L = 900 mm and D_h_ = 20 mm). The twisted tapes were inserted with the dimensions of (length = 20 mm, thickness = 0.5 mm, and profile = 30 mm). Meanwhile, the helical profile length, width, and path were 20 mm, 0.5 mm, and 30 mm. The twisted tape was tilted at an angle of 45° and 90°. Different working fluids such as DW, non-covalent nanofluids (GNPs-SDBS@DW), and covalent nanofluids (GNPs-COOH@DW) were tested inside the heat exchangers at T_in_ = 308 K, three different mass concentrations, and different Reynolds numbers. The outer walls of the spiral pipes were heated at a constant surface temperature of 330 K to examine the heat transfer enhancement parameters.

**Figure 1:**
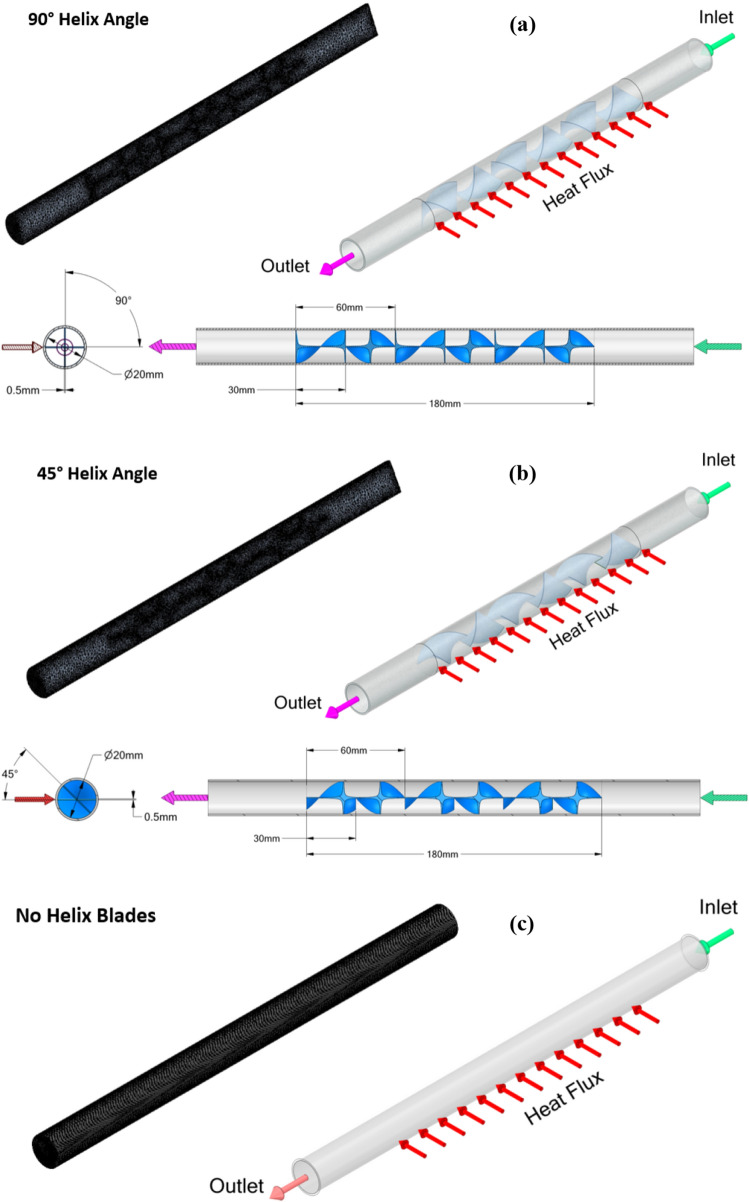
Schematic diagram of p physical and computational domain; (**a**) 90° helix angle, (**b**) 45° helix angle, (**c**) no helix blades.

Figure 1 illustrates a schematic design of the twisted tape insertion pipe with the applicable boundary conditions and grid domains. As noted, velocity and pressure boundary conditions are applied at the inlet and exit parts of the spiral pipes. The non-slip condition is applied to the pipe wall under the constant surface temperature. The pressure-based solution was used in the current numerical simulations. Meanwhile, (ANSYS FLUENT 2020R1) program was used to convert the partial differential equations (PDEs) into a system of algebraic equations using the finite volume method (FVM). The second-order SIMPLE (Semi-Implicit Method for Pressure Linked Equations-Consistent) methodology correlates the velocity–pressure. It should be emphasized that convergence for the residual of mass, momentum, and energy equations is less than 10^3^ and 10^6^, respectively.

### Assumptions and mathematical formulas

The homogeneous model is used to explain the nature of nanofluids. A continuous fluid with excellent thermophysical properties is formed by adding nanomaterials to the base fluid (DW). In this regard, the temperature and velocity of the base fluid and nanomaterials have the same values. The effective single-phase flow works due to the abovementioned theories and hypotheses in this research. Several examinations have confirmed the single-phase technique's validity and applicability for nanofluid flow^31,32^.

The flow of nanofluids is supposed to be Newtonian turbulent, incompressible, and steady-state. Compression work and viscous heating are not significant in this investigation. Also, the thicknesses of the inner and outer pipe walls are not considered. Therefore, the mass, momentum, and energy conservation governing equations of the thermal model may be stated as follows^33^:

Governing equation for mass

1 $$\nabla \cdot \left(\rho \overrightarrow{V}\right)=0$$

Governing equation for momentum

2 $$\nabla \cdot \left(\rho \overrightarrow{V}\overrightarrow{V}\right)=-\nabla P+\nabla \cdot \left(\left(u+{u}_{t}\right)\left(\nabla \overrightarrow{V}+\nabla {\overrightarrow{V}}^{T}\right)\right)+\rho \overrightarrow{g}$$

Governing equation for energy transport

3 $$\nabla \cdot \left(\rho {C}_{p}\overrightarrow{V}\right)=\nabla \cdot \left({k}_{eff}\nabla \mathrm{T}\right)+\rho \epsilon$$

where $\overrightarrow{V}$ is the mean velocity vector, K_eff_ = K + K_t_ is the effective thermal conductivity of covalent and non-covalent nanofluids, and ϵ is the energy dissipation rate. The effective thermo-physical properties of the nanofluid, including density (ρ), viscosity (μ), specific heat capacity (C_p_), and thermal conductivity (k) as measured in experimental study^34^ for a temperature of 308 K, as listed in Table 1 were used in these simulations.

**Table 1:** Thermal-physical properties of DW, (SDBS-GNPs@DW) and (COOH-GNPs@DW) nanofluids^34^.

Thermophysical properties	DW	SDBS-GNPs@DW			COOH-GNPs@DW		
		0.025 wt.%	0.05 wt.%	0.1 wt.%	0.025 wt.%	0.05 wt.%	0.1 wt.%
*ρ* (kg/m^3^)	994.1	995.1151	996.8895	998.158	994.9564	995.9241	996.8885
*cp* (J/kg·K)	4178	4152.66	4104.075	4055.95	4164.51	4111.11	4056.685
*k* (W/m·K)	0.623	0.645	0.675	0.695	0.685	0.71	0.754
*μ* (Ns/m^2^)	0.0007	0.001	0.0011	0.0013	0.0008	0.0009	0.0009

The turbulent nanofluid flow in plain and TT pipes was numerically simulated at the Reynolds numbers condition of 7000 ≤ Re ≤ 17,000. These simulation cases and the convective heat transfer coefficient were analyzed by applying the Mentor Shear Stress Transport (SST) κ-ω turbulence model, a two-equation Reynolds-averaged Navier–Stokes turbulence model that is commonly used for aerodynamic research. Moreover, this model operates with no wall functions and is accurate near-wall^35,36^. The (SST) κ-ω turbulence model governing equations are as follows:

Kinematic Eddy viscosity

4 $${\nu }_{T}=\frac{{a}_{1}k}{max\left({a}_{1}\omega ,S{F}_{2}\right)}$$

Turbulence kinetic energy

5 $$\frac{\partial k}{\partial t}+{U}_{j}\frac{\partial k}{\partial {x}_{j}}={P}_{k}-{\beta }^{*}k\omega +\frac{\partial }{\partial {x}_{j}}\left(\left(\nu +{\sigma }_{k}{\nu }_{T}\right)\frac{\partial k}{\partial {x}_{j}}\right)$$

Specific dissipation rate

6 $$\frac{\partial \omega }{\partial t}+{U}_{j}\frac{\partial \omega }{\partial {x}_{j}}=\alpha {S}^{2}-\beta {\omega }^{2}+\frac{\partial }{\partial {x}_{j}}\left(\left(\nu +{\sigma }_{\omega }{\nu }_{T}\right)\frac{\partial \omega }{\partial {x}_{j}}\right)+2\left(1-{F}_{1}\right){\sigma }_{{\omega }^{2}}\frac{1}{\omega }\frac{\partial k}{\partial {x}_{i}}\frac{\partial \omega }{\partial {x}_{i}}$$

Closure coefficients and auxiliary relations

7 $${F}_{2}=tanh\left(max{\left(\frac{2\sqrt{k}}{{\beta }^{*}\omega y},\frac{500\nu }{{y}^{2}\omega }\right)}^{2}\right)$$

8 $${P}_{k}=min\left({\tau }_{ij}\frac{\partial {U}_{i}}{\partial {x}_{i}},10{\beta }^{*}k\omega \right)$$

9 $${F}_{1}=tanh\left({\left(min\left(max\left(\frac{2\sqrt{k}}{{\beta }^{*}\omega y},\frac{500\nu }{{y}^{2}\omega }\right),\frac{4{\sigma }_{{\omega }^{2}}k}{C{D}_{k\omega }{y}^{2}}\right)\right)}^{4}\right)$$

10 $$C{D}_{k\omega }=max\left(2\rho {\sigma }_{{\omega }^{2}}\frac{1}{\omega }\frac{\partial k}{{\partial }_{{x}_{i}}}\frac{\partial \omega }{{\partial }_{{x}_{i}}},{10}^{-10}\right)$$

11 $$\phi ={\phi }_{1}{F}_{1}+{\phi }_{2}\left(1-{F}_{1}\right)$$

$${\alpha }_{1}=\frac{5}{9},{\alpha }_{2}=0.44$$

12 $${\beta }_{1}=\frac{3}{40},{\beta }_{2}=0.0828$$

$${\beta }^{*}=\frac{9}{100}$$

$${\sigma }_{{k}_{1}}=0.85,{\sigma }_{{k}_{2}}=1$$

$${\sigma }_{{\omega }_{1}}=0.5, {\sigma }_{{\omega }_{2}}=0.856$$

where $S$ is the strain rate magnitude and $y$ is the distance to the next surface. Meanwhile, ${\alpha }_{1}$, ${\alpha }_{2}$, ${\beta }_{1}$, ${\beta }_{2}$, ${\beta }^{*}$, ${\sigma }_{{k}_{1}}$, ${\sigma }_{{k}_{2}}$, ${\sigma }_{{\omega }_{1}}$ and ${\sigma }_{{\omega }_{2}}$ represent all model constants. F_1_ and F_2_ refer to the blending functions. Note: F_1_ = 1 inside the boundary layer and 0 in the free stream.

Performance evaluation parameters are used to examine turbulent convective heat transfer, covalent and non-covalent nanofluid flow, such as^31^:

Reynolds number

13 $$Re=\frac{\rho v{D}_{h}}{\mu }$$

Prandtl number

14 $$Pr=\frac{\mu {C}_{p}}{k}$$

Heat gain (W)

15 $${Q}_{Gain}=\dot{m}{\times C}_{p}\times \left({T}_{out}-{T}_{in}\right)$$

Heat transfer coefficient (W/m^2^. K)

16 $${h}_{tc}=\frac{{Q}_{Gain}}{{A}_{s}\times \left({\overline{T} }_{w}-{\overline{T} }_{f}\right)}$$

Average Nusselt number

17 $${Nu}_{avg}=\frac{{h}_{tc}{D}_{h}}{k}$$

Friction factor

18 $$f=\frac{2\times\Delta P\times {D}_{h}}{\rho \times {v}^{2}\times L}$$

Pressure loss

19 $$\Delta P={P}_{in}-{P}_{out}$$

Dittus–Boelter equation

20 $${Nu}_{avg}=0.023\times {Re}^{0.8}\times {Pr}^{0.4}$$

Petukhov equations

21 $$\begin{aligned}{Nu}_{avg}&=\frac{\left(\frac{f}{8}\right)RePr}{1.07+12.7{\left(\frac{f}{8}\right)}^{1/2}\left({Pr}^{2/3}-1\right)}\\ f&={\left(0.78\mathrm{ln}Re-1.64\right)}^{-2}\end{aligned}$$

Gnielinski equation

22 $${Nu}_{avg}=\frac{\left(\frac{f}{8}\right)(Re-1000)Pr}{1+12.7{\left(\frac{f}{8}\right)}^{1/2}\left({Pr}^{2/3}-1\right)}$$

Notter-Rouse equation

23 $${Nu}_{avg}=5+0.015{\times Re}^{0.856}\times {Pr}^{0.347}$$

Blasius equation

24 $$f=\frac{0.3164}{{Re}^{0.25}}$$

Thermohydraulic performance

25 $$PEC = \frac{{Nu_{{NFs}} }}{{Nu_{{DW}} }}/\left( {\frac{{f_{{NFs}} }}{{f_{{DW}} }}} \right)^{{1/3}} = \frac{{Nu_{{45^{^\circ } ,90^{^\circ } }} }}{{Nu_{{Plain}} }}/\left( {\frac{{f_{{45^{^\circ } ,90^{^\circ } }} }}{{f_{{Plain}} }}} \right)^{{1/3}} .$$

In this regard, ($\rho$), ($v$), (${D}_{h}$) and ($\mu$) are used for the density, working fluid velocity, hydraulic diameter, and dynamic viscosity. (${C}_{p}\, \mathrm{and}\, k$) are the specific heat capacity and thermal conductivity of the flowing fluid. Also, ($\dot{m}$) refer to the mass flow rate and (${T}_{out}-{T}_{in}$) symbolizes the outlet/inlet temperature difference. (NFs) refer to the covalent, non-covalent nanofluids, and (DW) refer to the distilled water (base fluid). ${A}_{s} = \pi DL$, ${\overline{T} }_{f}=\frac{\left({T}_{out}-{T}_{in}\right)}{2}$ and ${\overline{T} }_{w}=\sum \frac{{T}_{w}}{n}$.

## Thermal-physical properties of covalent and non-covalent nanofluids

The thermal-physical properties of base fluid (DW), non-covalent nanofluids (GNPs-SDBS@DW), and covalent nanofluids (GNPs-COOH@DW) were collected from the published literature (experimental study) under T_in_ = 308 K, as shown in Table 1^34^. In a typical experiment, to produce a non-covalent (GNP-SDBS@DW) nanofluid with known mass percentages, a certain gram of pristine GNPs was initially weighted via digital balance. A weight ratio SDBS/pristine GNPs of (0.5:1) suspended in DW. Meanwhile, the covalent (COOH-GNPs@DW) nanofluids were synthesized using a strong acid medium of HNO_3_ and H_2_SO_4_ in the volume ratio of (1:3) to add carboxyl groups at the surface of GNPs. The covalent and non-covalent nanofluids were suspended in DW with three different mass percentages, such as 0.025 wt.%, 0.05 wt.% and 0.1 wt.%.

## Results and discussion

### Verification of the numerical outputs

**Figure 2:**
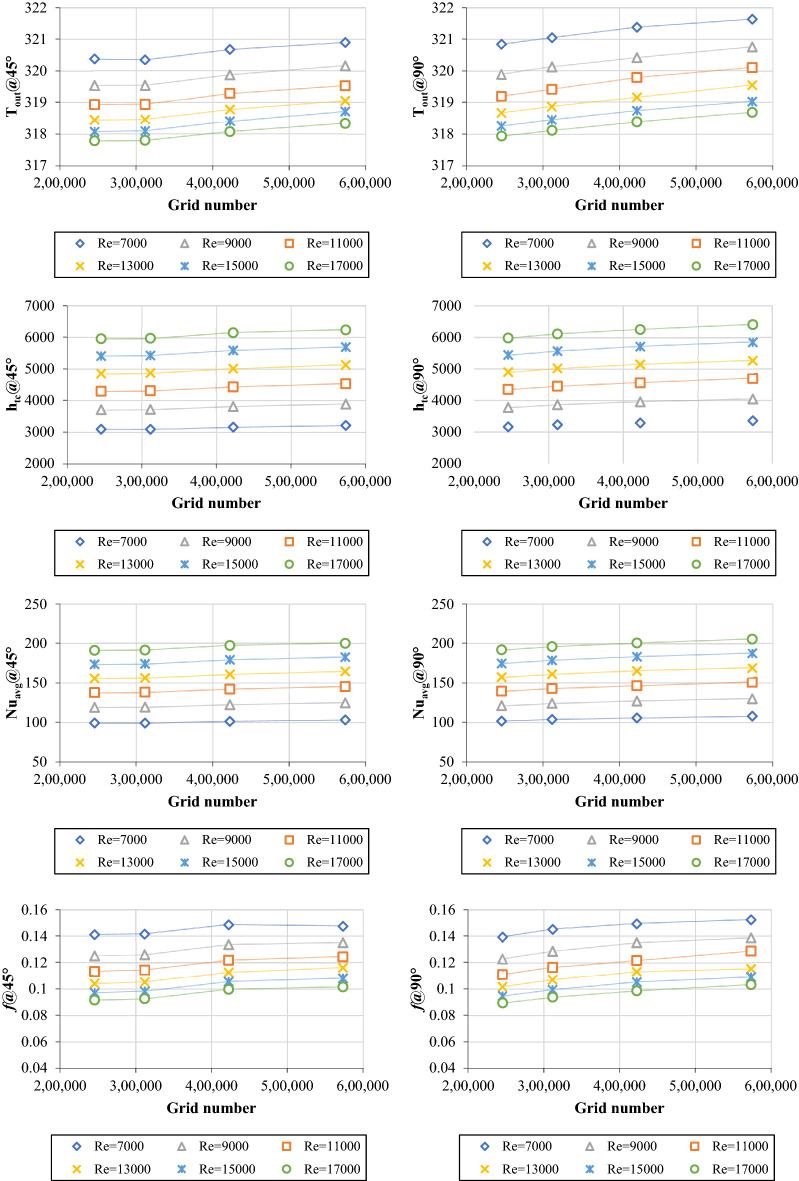

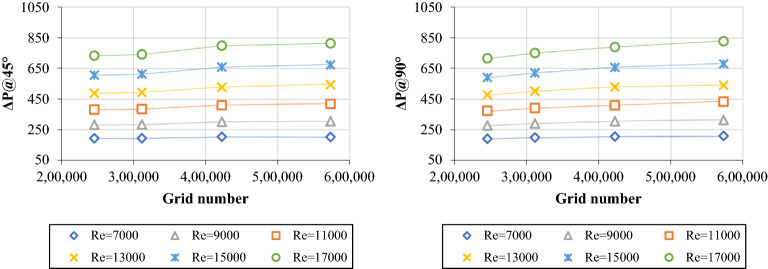
Grid independence tests for heat transfer and pressure drop properties using 45° and 90° twisted pipes for DW.

Grid independence tests were run on four different computational domains to ensure grid size did not affect the simulations. In the case of 45° twisted pipe, the number of elements was 249,033 for element size of 1.75 mm, 307,969 for element size of 2 mm, 421,406 for element size of 2.25 mm, and 564,940 for element size of 2.5 mm, respectively. Moreover, the number of elements in the instance of a 90° twisted pipe was 245,531 for element size of 1.75 mm, 311,584 for element size of 2 mm, 422,708 for element size of 2.25 mm, and 573,826 for element size of 2.5 mm, respectively. The accuracy of thermal properties such as (T_out_, h_tc,_ and Nu_avg_) readings increased by decreasing the number of elements. Meanwhile, the accuracy of friction factor and pressure drop values showed a completely different behavior (Fig. 2). Grid (2) was employed as the main mesh domain to evaluate thermohydraulic performance in the simulation cases.

**Figure 3:**
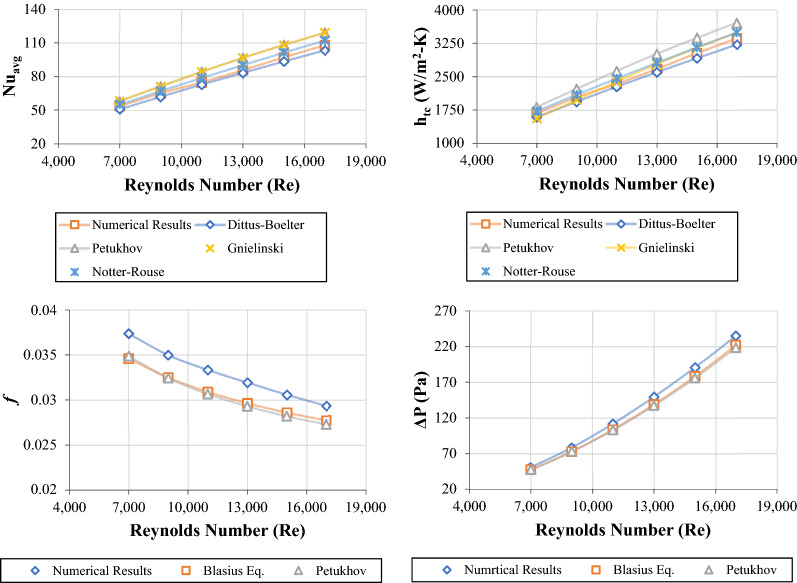
Heat transfer and fluid flow properties of DW at different Reynolds numbers using numerical calculations and empirical correlations.

The current numerical results were verified using well-known empirical correlations and equations such as Dittus–Boelter, Petukhov, Gnielinski, Notter-Rouse, and Blasius for heat transfer and friction factor properties. The comparison was under the condition of 7000 ≤ Re ≤ 17,000. As per Fig. 3, the average and maximum errors between the simulation results and heat transfer equations were 4.050% and 5.490% (Dittus–Boelter), 9.736% and 11.33% (Petukhov), 4.007% and 7.483% (Gnielinski), and 3.883% and 4.937% (Notter-Rouse). Meanwhile, the average and maximum errors between the simulation results and friction factor equations were 7.346% and 8.039% (Blasius) and 8.117% and 9.002% (Petukhov).

### Thermohydraulic enhancement of nanofluids/basefluids

**Figure 4:**
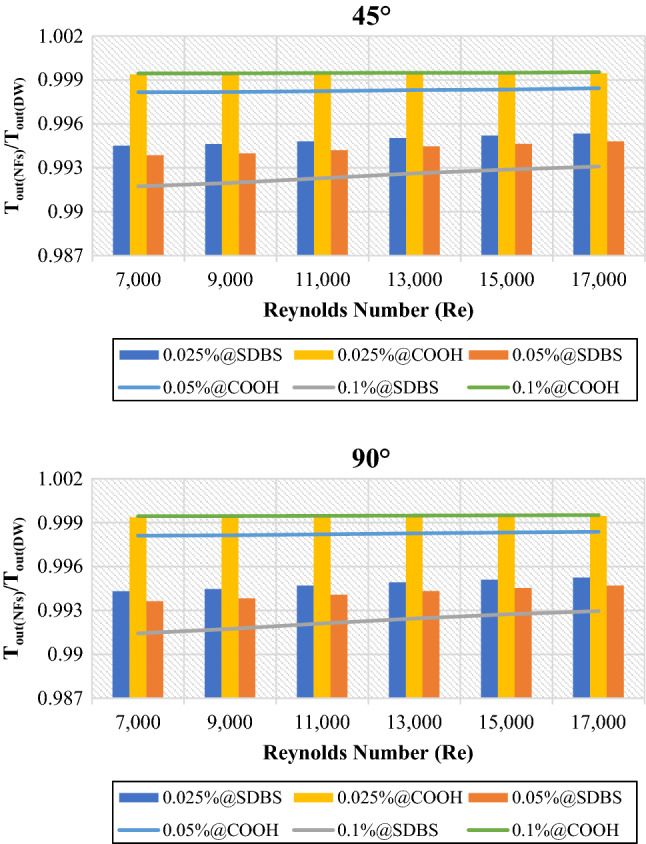
Outlet temperature of nanofluids to base fluid versus Reynolds numbers for 45° and 90° tubes.

This section discusses the thermohydraulic properties of non-covalent (GNPs-SDBS) and covalent (GNPs-COOH) based water nanofluids at three various mass fractions and Reynolds number as an average with respect to base fluid (DW). Two geometries twisted taped heat exchangers with (45° and 90° helix angles) were discussed in 7000 ≤ Re ≤ 17,000. Figure 4 shows the average outlet temperature of nanofluids to base fluid (DW) ($\frac{{{T}_{out}}_{NFs}}{{{T}_{out}}_{DW}}$) at (0.025 wt.%, 0.05 wt.% and 0.1 wt.%). ($\frac{{{T}_{out}}_{NFs}}{{{T}_{out}}_{DW}}$) is always less than 1, meaning that the outlet temperature of non-covalent (GNPs-SDBS) and covalent (GNPs-COOH) nanofluids was less than the outlet temperature for base fluid. The lowest and highest decrease was achieved by 0.1 wt.%-COOH@GNPs and 0.1 wt.%-SDBS@GNPs, respectively. This phenomenon is caused by an increase in the Reynolds number at the constant weight fraction, which causes a change in nanofluid characteristics (i.e., density and dynamic viscosity).

**Figure 5:**
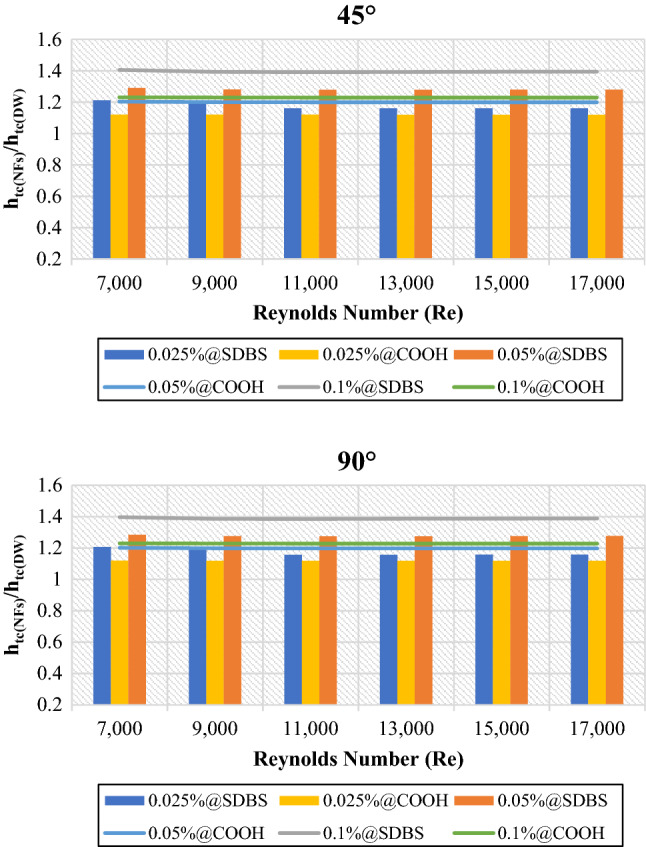
Heat transfer coefficient of nanofluids to base fluid versus Reynolds numbers for 45° and 90° tubes.

**Figure 6:**
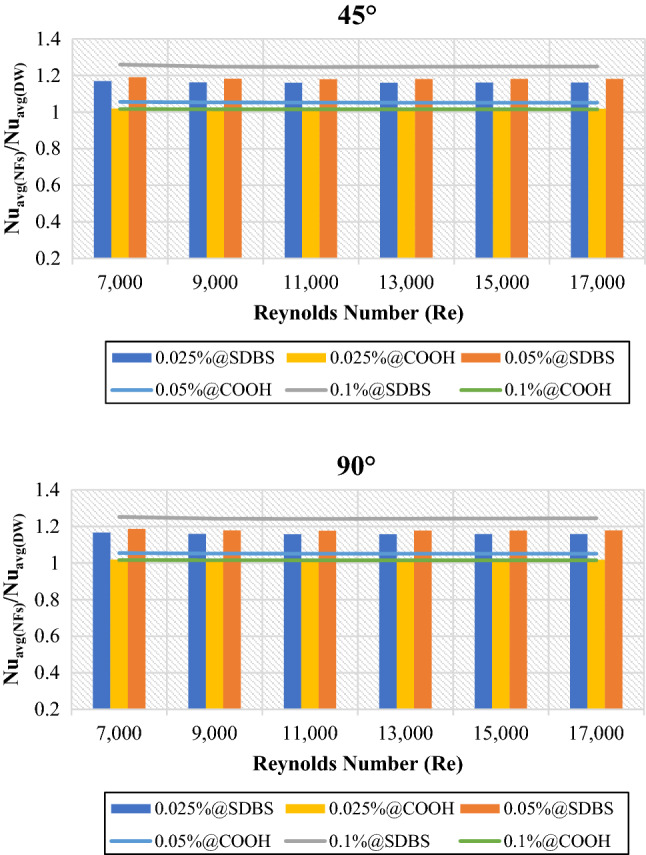
Average Nusselt number of nanofluids to base fluid versus Reynolds numbers for 45° and 90° tubes.

Figures 5 and 6 depict the average heat transfer properties of nanofluids to base fluid (DW) at (0.025 wt.%, 0.05 wt.% and 0.1 wt.%). The average heat transfer properties are always greater than one, meaning that the heat transfer properties of non-covalent (GNPs-SDBS) and covalent (GNPs-COOH) nanofluids were enhanced relative to the base fluid. The lowest and highest enhancement was achieved by 0.1 wt.%-COOH@GNPs and 0.1 wt.%-SDBS@GNPs, respectively. Heat transport properties improved when the Reynolds number increased due to greater fluid mixing and turbulence in tube^1^. The liquid running through the small gaps gets a higher velocity, causing velocity/thermal boundary layers to thin, thus, enhancing the heat transfer rate. Adding more nanoparticle percentages to the base fluid exhibits positive and negative outcomes. The favorable influences include increased nanoparticle collision, fluid thermal conductivity, and beneficial requirements for heat transfer augmentation.

Meanwhile, the negative impact is the increased dynamic viscosity of nanofluid, which decreases the movement of nanofluid and, therefore, the average Nusselt number (Nu_avg_). The increased thermal conductivity of the (GNPs-SDBS@DW) and (GNPs-COOH@DW) nanofluids is supposed to be due to Brownian motion and micro-convection of graphene nanoparticles suspended in DW^37^. (GNPs-COOH@DW) nanofluids had a higher thermal conductivity than (GNPs-SDBS@DW) nanofluids and distilled water. Adding more nanomaterial percentages to the base fluid increased their thermal conductivity (Table 1)^38^.

**Figure 7:**
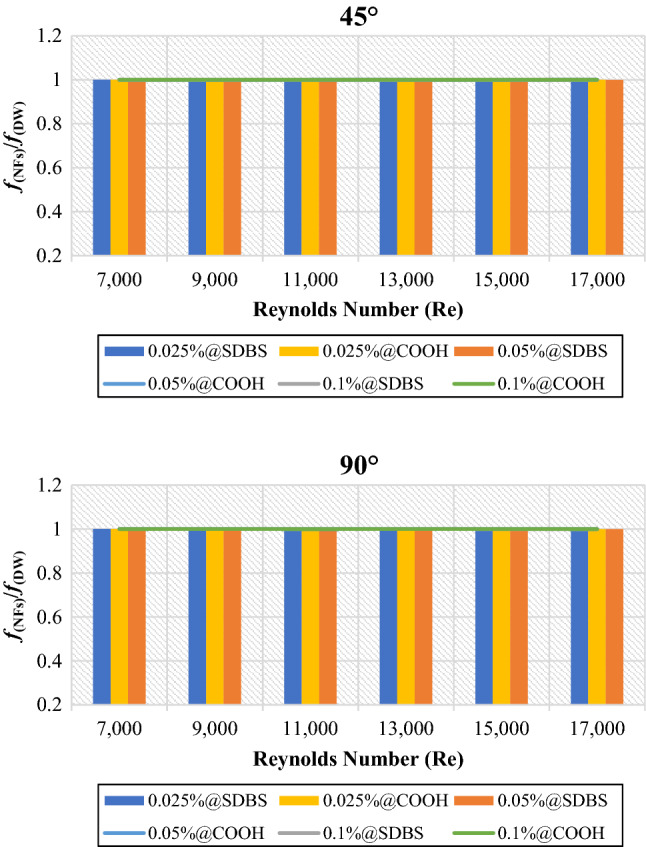
Friction factor of nanofluids to base fluid versus Reynolds numbers for 45° and 90° tubes.

Figure 7 explains the average friction factor of nanofluids to base fluid (DW) (*f*(_NFs_)/*f*(_DW_)) at the mass percentages of (0.025%, 0.05% and 0.1%). The average friction factor is always **≈** 1, implying that the friction factor of non-covalent (GNPs-SDBS@DW) and covalent (GNPs-COOH@DW) nanofluids was the same with base fluid. The heat exchangers with less space created more flow obstruction and increased flow friction^1^. Mainly, the friction factor increased marginally along with increasing nanofluid mass percentages. The higher friction loss was caused by increased nanofluid dynamic viscosity and shear stresses on surfaces with higher nano-graphene mass percentages in the base fluid. According to Table (1), the dynamic viscosity of the (GNPs-SDBS@DW) nanofluid was higher than that of the (GNPs-COOH@DW) nanofluid at equal weight percentages, because of the impact of adding surfactant during the production of non-covalent nanofluids.

**Figure 8:**
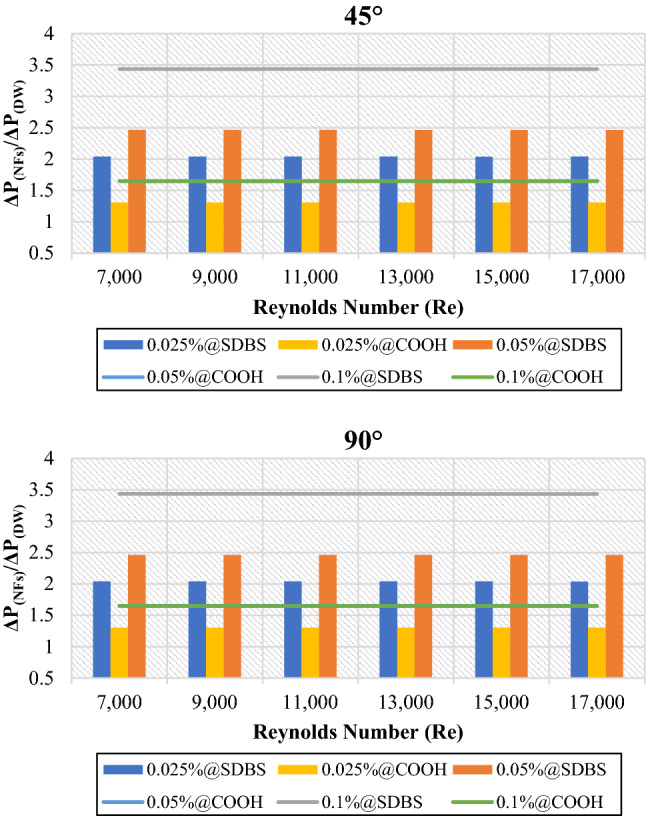
Pressure drops of nanofluids to base fluid versus Reynolds numbers for 45° and 90° tubes.

Figure 8 shows the average pressure loss of nanofluids to base fluid (DW) ($\frac{{\Delta P}_{NFs}}{{\Delta P}_{DW}}$) at the mass percentages of (0.025%, 0.05% and 0.1%). Non-covalent (GNPs-SDBS@DW) nanofluids demonstrate higher average pressure loss and increase by increasing the weight percentage to 2.04% for 0.025 wt.%, 2.46% for 0.05 wt.%, and 3.44% for 0.1 wt.% in both cases (45° and 90° helix angles). Meanwhile, (GNPs-COOH@DW) nanofluids exhibited lower average pressure loss, increasing from 1.31% for 0.025 wt.% to 1.65% for 0.05 wt.%. The average pressure loss for 0.05 wt.%-COOH@GNPs and 0.1 wt.%-COOH@GNPs is 1.65%. As shown, the pressure drop increased in all cases by the Re number increment. The increased pressure drop in high Re values could be justified by the direct relation with the volume flow rate. Therefore, higher Re numbers in tubes bring about a higher pressure drop, which calls for the increased pumping power^39,40^. Moreover, higher pressure loss because of higher swirl and turbulence intensities produced by the larger surface area increased the interaction of pressure forces with inertial forces in the boundary layer^1^.

**Figure 9:**
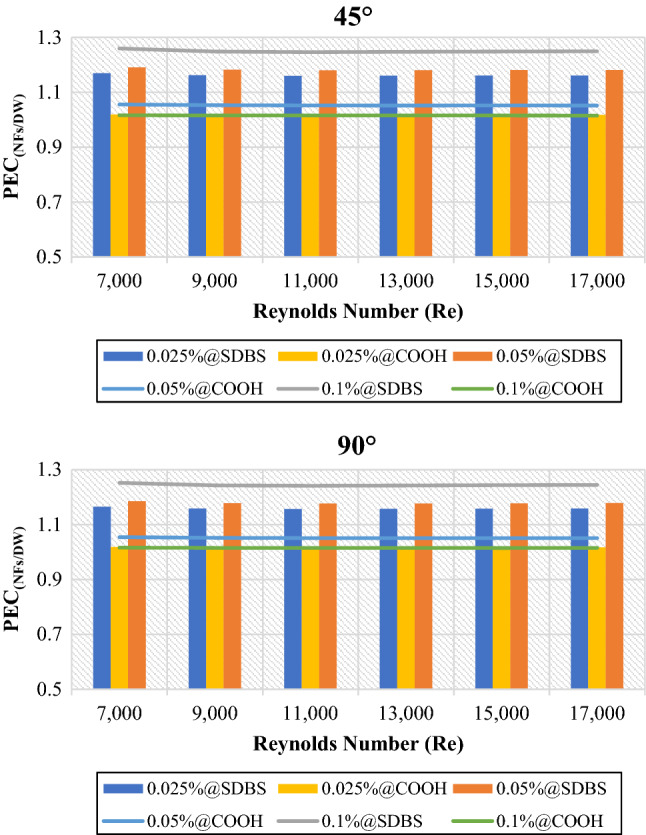
Hydrothermal performance of nanofluids to base fluid versus Reynolds numbers for 45° and 90° tubes.

Overall, the performance evaluation criterion (PEC) of non-covalent (GNPs-SDBS@DW) and covalent (GNPs-COOH@DW) nanofluids is shown in Fig. 9. (GNPs-SDBS@DW) nanofluids present higher PEC values than (GNPs-COOH@DW) in both cases (45° and 90° helix angles) and are raised by increasing the mass fractions such as 1.17 for 0.025 wt.%, 1.19 for 0.05 wt.% and 1.26 for 0.1 wt.%. Meanwhile, the value of PEC using (GNPs-COOH@DW) nanofluids is 1.02 for 0.025 wt.%, 1.05 for 0.05 wt.% and 1.02 for 0.1 wt.% in both cases (45° and 90° helix angles). Generally, as the Reynolds number increased, the thermohydraulic performance factor decreased considerably. The drop in thermohydraulic performance factor systematically is credited to the rise of (Nu_NFs_/Nu_DW_) and the decrease of (*f*_NFs_/*f*_DW_) as the Reynolds number increases^1^.

### Thermohydraulic enhancement of twisted pipe/plain pipe

**Figure 10:**
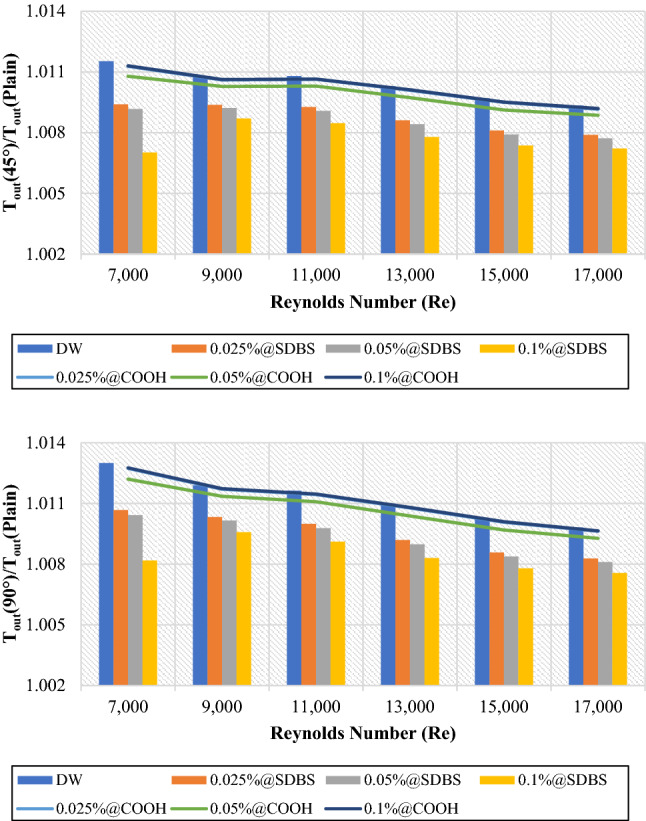
Outlet temperature of (45° and 90° helix angles) relative to plain pipe versus Reynolds numbers for different nanofluids.

**Figure 11:**
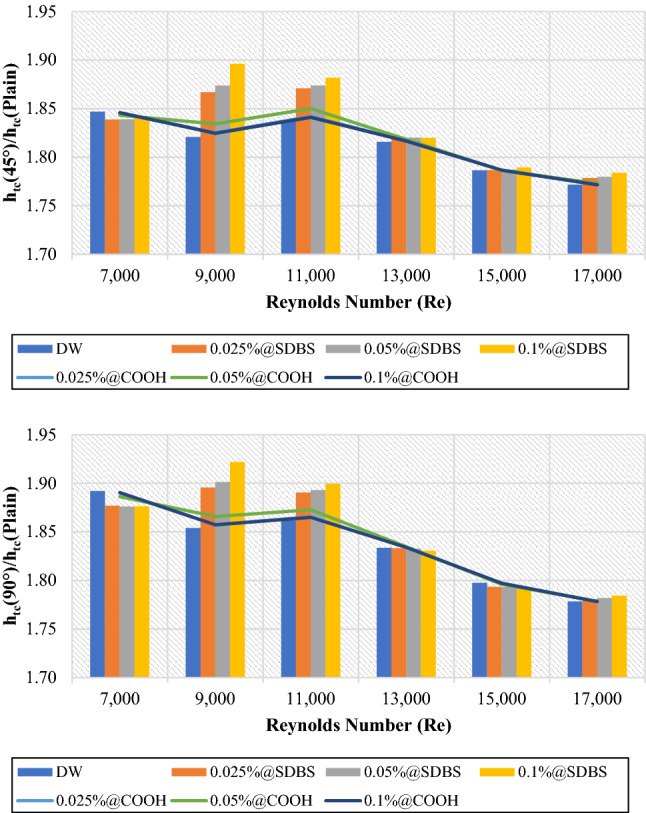
Heat transfer coefficient of (45° and 90° helix angles) relative to plain pipe versus Reynolds numbers for different nanofluids.

This section discusses the thermohydraulic properties of water (DW), non-covalent (GNPs-SDBS@DW), and covalent (GNPs-COOH@DW) nanofluids at three different weight concentrations and Reynolds numbers. Two twisted taped heat exchanger geometries were considered with (45° and 90° helix angles) in the range of 7000 ≤ Re ≤ 17,000 relatives to plain pipe to evaluate the average values of thermohydraulic properties. Figure 10 shows the outlet temperature of water and nanofluids as an average value using (45° and 90° helix angles) to the plain pipe ($\frac{{{T}_{out}}_{Twisted}}{{{T}_{out}}_{Plain}}$). The non-covalent (GNPs-SDBS@DW) and covalent (GNPs-COOH@DW) nanofluids were in three different mass fractions, such as 0.025 wt.%, 0.05 wt.% and 0.1 wt.%. As illustrated in Fig. 11, the average outlet temperature values ($\frac{{{T}_{out}}_{Twisted}}{{{T}_{out}}_{Plain}}$) are > 1, indicating that the outlet temperature of (45° and 90° helix angles) heat exchangers was more significant than the value of outlet temperature for the plain pipe due to a more vigorous turbulence intensity and better fluid mixing. Furthermore, as the Reynolds number rises, the outlet temperature of DW, non-covalent, and covalent nanofluids declines. Based fluid (DW) has the highest average output temperature values. Meanwhile, the lowest value is dedicated for 0.1 wt.%-SDBS@GNPs. The non-covalent (GNPs-SDBS@DW) nanofluids show lower average outlet temperature relative to covalent (GNPs-COOH@DW) nanofluids. As the flow field is mixed up more as a result of the twisted tape, the wall heat flux can more easily pass through the fluid flow, raising the bulk temperature. Smaller twisted tape ratio values result in better penetration, which improves heat transmission. The twisted tape, on the other hand, is seen to maintain a lower temperature near the wall, which in turn raises Nu_avg_. With twisted tape inserts, a higher Nu_avg_ indicates improved convective heat transmission across tube^22^. Increased residence time due to raised flow path with extra mixing and turbulence creation, they are resulting in a rise in the fluid's outlet temperature^41^.

**Figure 12:**
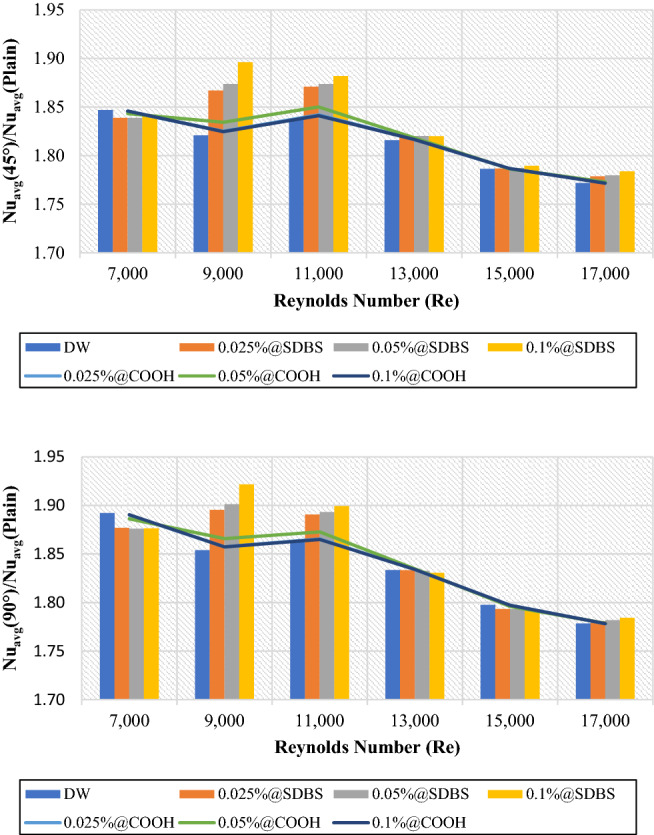
Average Nusselt number of (45° and 90° helix angles) relative to plain pipe versus Reynolds numbers for different nanofluids.

The primary mechanisms of heat transfer enhancement owing to twisted tape are as follows: 1. The lowering of a heat transfer tube's hydraulic diameter generates an increase in flow velocity and curvature, which in turn increases shear stress near the wall and promotes secondary motion. 2. The velocity increases near the tube wall due to the blocked twisted tape, reducing the thickness of the boundary layer. 3. The helical flow following the twisted tape causes an increase in velocity. 4. Induced whirling flow improves fluid mixing between the core and near-wall flow areas^42^. Figures 11 and 12 showed the heat transfer properties such as (heat transfer coefficient and average Nusselt number) of DW and nanofluids as an average value using twisted tape inserts pipes relative to the plain pipe. The non-covalent (GNPs-SDBS@DW) and covalent (GNPs-COOH@DW) nanofluids were in three different mass fractions, such as 0.025 wt.%, 0.05 wt.% and 0.1 wt.%. In both heat exchangers (45° and 90° helix angles), the average values of heat transfer properties are > 1, which indicates improvement of heat transfer coefficient and average Nusselt number using twisted pipes relative to plain pipe. The non-covalent (GNPs-SDBS@DW) nanofluids show higher average heat transfer enhancement than covalent (GNPs-COOH@DW) nanofluids. The highest augmentation in the heat transfer properties was reached by 0.1 wt.%-SDBS@GNPs with the value of 1.90 in both heat exchangers (45° and 90° helix angles) at Re = 900. This means that the role of uniform TT in increasing turbulence intensity is far more major at the lower fluid velocities (Reynolds numbers)^43^. The heat transfer coefficient and average Nusselt number in TT pipes are higher than in a plain pipe due to the induction of multiple swirl flows, resulting in thinner boundary layer. Comparison to the basic pipe (no twisted tape insertions), whether the existence of TT produces increased turbulence intensity, flow mixing of working fluids, and heat transfer enhancement^21^.

**Figure 13:**
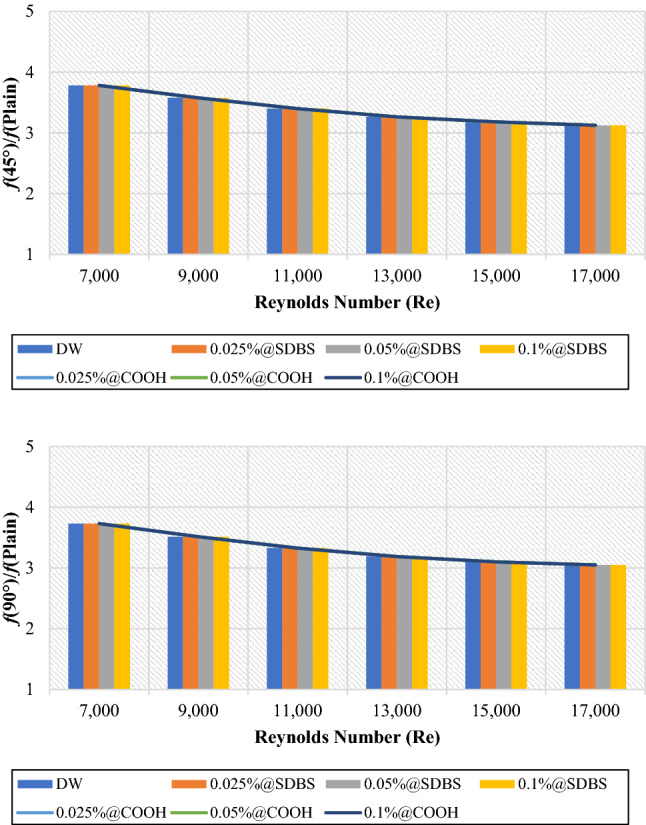
Friction factor of (45° and 90° helix angles) relative to plain pipe versus Reynolds numbers for different nanofluids.

**Figure 14:**
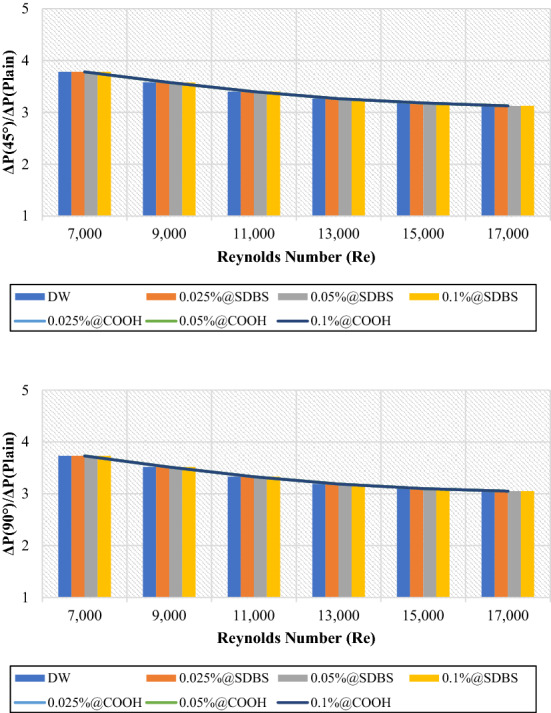
Pressure loss of (45° and 90° helix angles) relative to plain pipe versus Reynolds numbers for different nanofluids.

Figures 13 and 14 presented the average friction factor ($\frac{{f}_{Twisted}}{{f}_{Plain}}$) and pressure loss ($\frac{{\Delta P}_{Twisted}}{{\Delta P}_{Plain}}$) of 45° and 90° heat exchangers relative to the plain pipe using DW, (GNPs-SDBS@DW) and (GNPs-COOH@DW) nanofluids with (0.025 wt.%, 0.05 wt.%, and 0.1 wt.%). It can be observed from Figs. 13 and 14, as the Reynolds number increases in both heat exchangers (45° and 90° helix angles), the ratio of friction factor ($\frac{{f}_{Twisted}}{{f}_{Plain}}$) and pressure loss ($\frac{{\Delta P}_{Twisted}}{{\Delta P}_{Plain}}$) decreases. For all scenarios evaluated, the friction factor and pressure loss values are superior at lower Reynolds numbers. The average friction factor and pressure loss are between 3.78 and 3.12. The average friction factor and pressure loss show that the value of (45° and 90° helix angles) heat exchangers has risen three times than the plain pipe. Furthermore, by flowing higher working fluid velocity, the friction factor decreases. This problem is because, by raising the Reynolds number, the thickness of the boundary layer decreases, causing reducing in the influence of the affected area by dynamic viscosity and decreasing velocity gradient and shear stress, and, therefore, reducing the friction factor^21^. Improvement of the blocking effect because of the existence of TT and increased swirling flows produces a much higher-pressure loss for the non-uniform TT pipe than the basic ones. Additionally, for both the basic and TT pipes, it can be seen that the pressure drop is increasing by increasing the velocity of the working fluids^43^.

**Figure 15:**
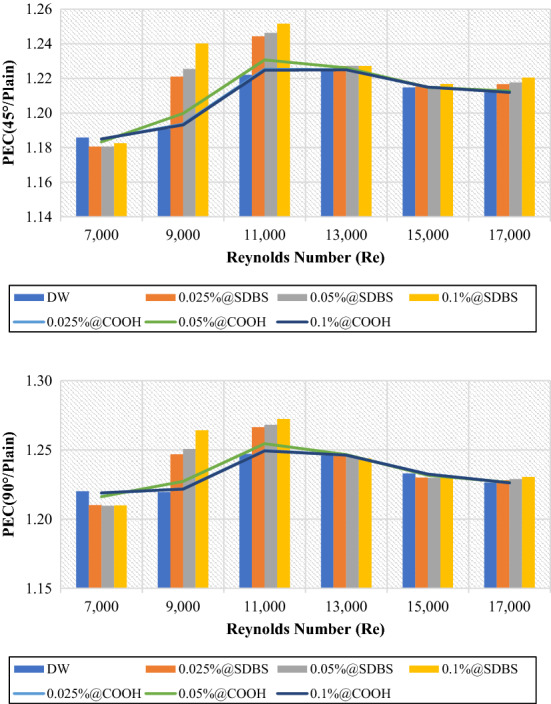
Performance evaluation criterion of (45° and 90° helix angles) relative to plain pipe versus Reynolds numbers for different nanofluids.

Generally, Fig. 15 illustrates the performance evaluation criterion (PEC) of 45° and 90° heat exchangers relative to the plain pipe ($\frac{{PEC}_{Twisted}}{{PEC}_{Plain}}$) using DW, (GNPs-SDBS@DW) and covalent (GNPs-COOH@DW) nanofluids in (0.025 wt.%, 0.05 wt.%, and 0.1 wt.%). The value for the ($\frac{{PEC}_{Twisted}}{{PEC}_{Plain}}$) is > 1 in both instances (45° and 90° helix angles) heat exchangers. Furthermore, the better value of ($\frac{{PEC}_{Twisted}}{{PEC}_{Plain}}$) is reached at Re = 11,000. The 90°-degree angle heat exchanger revealed a modest increase ($\frac{{PEC}_{Twisted}}{{PEC}_{Plain}}$) values in comparison to the 45°-degree angle heat exchanger. Furthermore, at Re = 11,000, 0.1 wt.%-GNPs@SDBS indicates a higher ($\frac{{PEC}_{Twisted}}{{PEC}_{Plain}}$) value, such as 1.25 for 45°-degree angle heat exchanger and 1.27 for 90°-degree angle heat exchanger. It is larger than unity at all mass fraction percentages, pointing out that the pipe with twisted tape inserts outperforms the plain pipe. It is noted that heat transfer augmentation supplied by the tape inserts results in significantly increased friction loss^22^.

### Velocity contours and streamlines

Appendix A displays the velocity streamlines of 45° and 90° heat exchangers that use the DW, 0.1 wt.%-GNPs-SDBS@DW, and 0.1 wt.%-GNPs-COOH@DW at Re = 7000. The streamlines in the transverse planes are the most remarkable features of the impact of the twisted tape inserts on the mainstream flow. The applications of 45° and 90° heat exchangers illustrated about the same velocity in the near-wall regions. Meanwhile, Appendix B illustrates the velocity contours of 45° and 90° heat exchangers using DW, 0.1 wt.%-GNPs-SDBS@DW, and 0.1 wt.%-GNPs-COOH@DW at Re = 7000. The velocity contours were at three separate locations (slices) such as Plain-1 (P1 = −30 mm), Plain-4 (P4 = 60 mm) and Plain-7 (P7 = 150 mm). The lowest velocity is near the pipe wall, and fluid velocity increases in the direction of the pipe center. Also, moving across the pipe increases the low-velocity zones next to the wall. This is because of the growth of hydrodynamic boundary layers, which increases the thickness of the low velocity zone next to the wall. Moreover, increasing the Reynolds number improves the total velocity level at all cross-sections, reducing the thickness of the low-velocity zones through pipe^39^.

## Conclusions

Covalent and non-covalent functionalized Graphene nanoplatelets were evaluated inside twisted tape inserts with 45° and 90° helix angles. The heat exchangers were numerically solved via SST k-omega turbulence models in 7000 ≤ Re ≤ 17,000. The thermophysical properties were calculated at T_in_ = 308 K. At the same time, the walls of the twisted pipe were heated under a constant temperature of 330 K. (GNPs-SDBS@DW) and (GNPs-COOH@DW) nanofluids in three mass dilutions such as (0.025 wt.%, 0.05 wt.% and 0.1 wt.%). Current research considers six principal factors: outlet temperature, heat transfer coefficient, average Nusselt number, friction factor, pressure loss, and performance evaluation criterion. The following are the key findings:

i. The average outlet temperature (${{T}_{out}}_{Nanofluids}$/${{T}_{out}}_{Basefluid}$) is always less than 1, meaning that the outlet temperature of non-covalent (GNPs-SDBS@DW) and covalent (GNPs-COOH@DW) nanofluids was less than of outlet temperature for base fluid. Meanwhile, the average outlet temperature (${{T}_{out}}_{Twisted}$/${{T}_{out}}_{Plain}$) values are > 1, indicating that the outlet temperature of (45° and 90° helix angles) was more substantial than the value of outlet temperature for the plain pipe.
ii. In both cases, the average (Nanofluids/Basefluids) and (Twisted pipe/Plain pipe) of heat transfer properties always show > 1. The non-covalent (GNPs-SDBS@DW) nanofluids showed higher average heat transfer augmentation corresponding to covalent (GNPs-COOH@DW) nanofluids.
iii. The average friction factor (${f}_{Nanofluids}/{f}_{Basefluid}$) of non-covalent (GNPs-SDBS@DW) and covalent (GNPs-COOH@DW) nanofluids is always **≈** 1. Meanwhile, the average friction factor (${f}_{Twisted}/{f}_{Plain}$) of non-covalent (GNPs-SDBS@DW) and covalent (GNPs-COOH@DW) nanofluids is always > 3.
iv. In both cases (45° and 90° helix angles), (GNPs-SDBS@DW) nanofluids showed higher (${\Delta P}_{Nanofluids}/{\Delta P}_{Basefluid}$) as 2.04% for 0.025 wt.%, 2.46% for 0.05 wt.% and 3.44% for 0.1 wt.%. In the meantime, (GNPs-COOH@DW) nanofluids showed lower (${\Delta P}_{Nanofluids}/{\Delta P}_{Basefluid}$) from 1.31% for 0.025 wt.% to 1.65% for 0.05 wt.%. Furthermore, the average pressure loss (${\Delta P}_{Twisted}/{\Delta P}_{Plain}$) of non-covalent (GNPs-SDBS@DW) and covalent (GNPs-COOH@DW) nanofluids is always > 3.
v. In both cases (45° and 90° helix angles), (GNPs-SDBS@DW) nanofluids presented higher (${PEC}_{Nanofluids}/{PEC}_{Basefluid}$) values than (GNPs-COOH@DW), such as 1.17 for 0.025 wt.%, 1.19 for 0.05 wt.% and 1.26 for 0.1 wt.%. Meanwhile, the value of (${PEC}_{Nanofluids}/{PEC}_{Basefluid}$) using (GNPs-COOH@DW) nanofluids was 1.02 for 0.025 wt.%, 1.05 for 0.05 wt.% and 1.02 for 0.1 wt.%. Moreover, at Re = 11,000, 0.1 wt.%-GNPs@SDBS shows the higher (${PEC}_{Twisted}/{PEC}_{Plain}$) value, such as 1.25 for 45° helix angle and 1.27 for 90° helix angle.

## Data Availability

All the data generated or analyzed during the current study are included in this published article.

## Appendix A

Velocity streamlines of 45° and 90° heat exchangers using DW, 0.1wt.%-GNPs-SDBS@DW and 0.1wt.%-GNPs-COOH@DW at Re = 7000.

## Appendix B

Velocity contours of 45° and 90° heat exchangers using DW, 0.1wt.%-GNPs-SDBS@DW and 0.1wt.%-GNPs-COOH@DW at Re = 7000.

## List of symbols

Ag: Silver
Al_2_O_3_: Aluminum oxide
*A*_*s*_: Surface area of tube (m^2^)
CMC: Carboxymethyl cellulose
COOH: Carboxylic acid
*C*_*p*_: Specific heat capacity (J/kg·K)
Cu: Copper
CuO: Copper oxide
*D*_*h*_: Tube hydraulic diameter (mm)
DTTI: Dimpled twisted turbulator insert
DW: Distilled water
*f*: Friction factor
Fe: Iron
FVM: Finite volume method
GNPs: Graphene nanoplatelets
GO: Graphene oxide
Gr: Graphene
H/D: Twisted tape ratio
H_2_SO_4_: Sulfuric acid
HNO_3_: Nitric acid
*h*_*tc*_: Heat transfer coefficient (W/m^2^. K)
*k*: Thermal conductivity (W/m·K)
*k*_*eff*_: Effective thermal conductivity (W/m K)
*L*: Tube length (mm)
$\dot{m}$: Mass flow rate (kg/s)
MgO: Magnesium oxide
MWCNTs: Multi-walled carbon nanotubes
*Nu*_*avg*_: Average Nusselt number
PCM: Phase change material
PEC: Performance assessment criteria
Pr: Prandtl number
Pt: Platinum
*Q*_*gain*_: Heat gain (W)
Re: Reynolds number
SDBS: Sodium dodecylbenzenesulfonate
SST: Spiky twisted tapes
*T*_*f*_: Bulk temperature (K)
THNF: Tripartite hybrid nanofluids
*T*_*in*_: Inlet fluid temperature (K)
*TiO*_2_: Titanium dioxide
*T*_*out*_: Outlet fluid temperature (K)
*T*_*w*_: Wall surface temperature (K)
*v*: Working fluid velocity (m/s)
VcTT: V-cuts twisted tape
WC: Wire coil
wt.%: Weight concentration of nanoparticles
*ΔP*: Pressure drop (Pa)
*ϵ*: Energy dissipation rate (m^2^/s^3^)
*μ*: Dynamic viscosity (Ns/m^2^)
*ρ*: Density (kg/m^3^)
